# Lessons learned: Development of COVID-19 clinical staging models at a large urban research institution

**DOI:** 10.1017/cts.2023.26

**Published:** 2023-03-27

**Authors:** Sean S. Huang, Lelia H. Chaisson, William Galanter, Arash Jalali, Martha Menchaca, Natalie Parde, Jorge M. Rodríguez-Fernández, Andrew Trotter, Karl M. Kochendorfer

**Affiliations:** 1 Department of Medicine, Division of Academic Internal Medicine and Geriatrics, University of Illinois Chicago, Chicago, IL, USA; 2 Department of Medicine, Division of Infectious Diseases, University of Illinois Chicago, Chicago, IL, USA; 3 Center for Global Health, University of Illinois Chicago, Chicago, IL, USA; 4 Department of Pharmacy Systems, Outcomes, and Policy, University of Illinois Chicago, Chicago, IL, USA; 5 School of Public Health, University of Illinois Chicago, Chicago, IL, USA; 6 Department of Radiology, University of Illinois Chicago, Chicago, IL, USA; 7 Department of Computer Science, University of Illinois Chicago, Chicago, IL, USA; 8 Department of Neurology and Rehabilitation, University of Illinois Chicago, Chicago, IL, USA; 9 Department of Family and Community Medicine, University of Illinois Chicago, Chicago, IL, USA

**Keywords:** Lessons learned, COVID-19, collaboration, data infrastructure, data acquisition, data validation, predictive models, translation, information systems

## Abstract

**Background/Objective:**

The University of Illinois at Chicago (UIC), along with many academic institutions worldwide, made significant efforts to address the many challenges presented during the COVID-19 pandemic by developing clinical staging and predictive models. Data from patients with a clinical encounter at UIC from July 1, 2019 to March 30, 2022 were abstracted from the electronic health record and stored in the UIC Center for Clinical and Translational Science Clinical Research Data Warehouse, prior to data analysis. While we saw some success, there were many failures along the way. For this paper, we wanted to discuss some of these obstacles and many of the lessons learned from the journey.

**Methods:**

Principle investigators, research staff, and other project team members were invited to complete an anonymous Qualtrics survey to reflect on the project. The survey included open-ended questions centering on participants’ opinions about the project, including whether project goals were met, project successes, project failures, and areas that could have been improved. We then identified themes among the results.

**Results:**

Nine project team members (out of 30 members contacted) completed the survey. The responders were anonymous. The survey responses were grouped into four key themes: Collaboration, Infrastructure, Data Acquisition/Validation, and Model Building.

**Conclusion:**

Through our COVID-19 research efforts, the team learned about our strengths and deficiencies. We continue to work to improve our research and data translation capabilities.

## Introduction

The University of Illinois at Chicago (UIC), along with many academic institutions worldwide, made significant efforts to address the many challenges presented by the COVID-19 pandemic. Many institutions leveraged electronic medical information [1–3] to develop COVID-19 risk models. Unfortunately, few models demonstrated clinical utility [4–6]. Deploying novel models in hospital settings is often complicated and requires robust infrastructure and innovation [7] as machine learning shifts from development to deployment. Our goal was to define a COVID-19 clinical staging system to stratify patient risk and provide an accurate prognosis based on demographics, medical history, medications, symptoms, physical exam findings, laboratory results, and imaging findings. The rationale was that this system would help our institution improve patient care and hospital preparedness.

Data from patients with a clinical encounter at UIC from July 1, 2019 to March 30, 2022 were abstracted from the University of Illinois electronic health record (EHR) and stored in the UIC Center for Clinical and Translational Science (CCTS) Clinical Research Data Warehouse (CRDW). We included patients with confirmed COVID-19, and compared them to patients without COVID-19 admitted within the same time frame. Radiology images were collected by UI Health Picture Archiving and Communication System (PACS). Analyses were conducted within a secure computing environment (SCE). A CCTS COVID-19 Rapid Response Pilot grant was obtained to support this project.

This project provided an opportunity for collaboration between physicians, researchers, informaticists, and engineers across the University of Illinois System, including the Chicago and Urbana-Champaign campuses. This represented a novel initiative. We hoped to strengthen both institutions’ informatics and data science capabilities with this collaboration. In addition, we aimed to improve our computing and data infrastructure for performing research. We also hoped to improve the agility and timeliness of our university and health system’s response to urgent clinical research questions and their direct application to a novel and evolving health problem. This capability would be critically important in a time when globalization and other factors have made emerging infectious diseases an ongoing public health issue [8].

During this project, our institution employed techniques to extract relevant information from unstructured UI Health data and gleaned many insights about COVID-19 patients hospitalized at UIC. In addition, we generated multiple predictive models, incorporated machine learning, leveraged natural language processing (NLP), and performed imaging analytics. Our work has been published in several peer-reviewed journals [9–13]. New goals and aims were developed throughout the project, including examining social determinants of health and comparing our findings with larger national and regional COVID-19 patient datasets (e.g., NC3 [14]).

However, there were many challenges and failures along the way. For instance, we had a few rejected grants, including an NIH NLM R21 grant and at least three others centered around artificial intelligence. When we did receive funding, we often had difficulty finding approval to utilize those funds. The collaboration between UIC and UIUC could have been more seamless and required the melding of disparate systems. There were bandwidth challenges among many team members, leading to slowdowns. We also had descriptive analyses that were not able to be completed as key members transitioned to operations. An expensive SCE led to distress and unforeseen technical challenges for our research staff. There were many scope challenges as well. This paper discusses many of these obstacles and more while reflecting on the lessons we have learned.

## Methods

**Figure 1.**
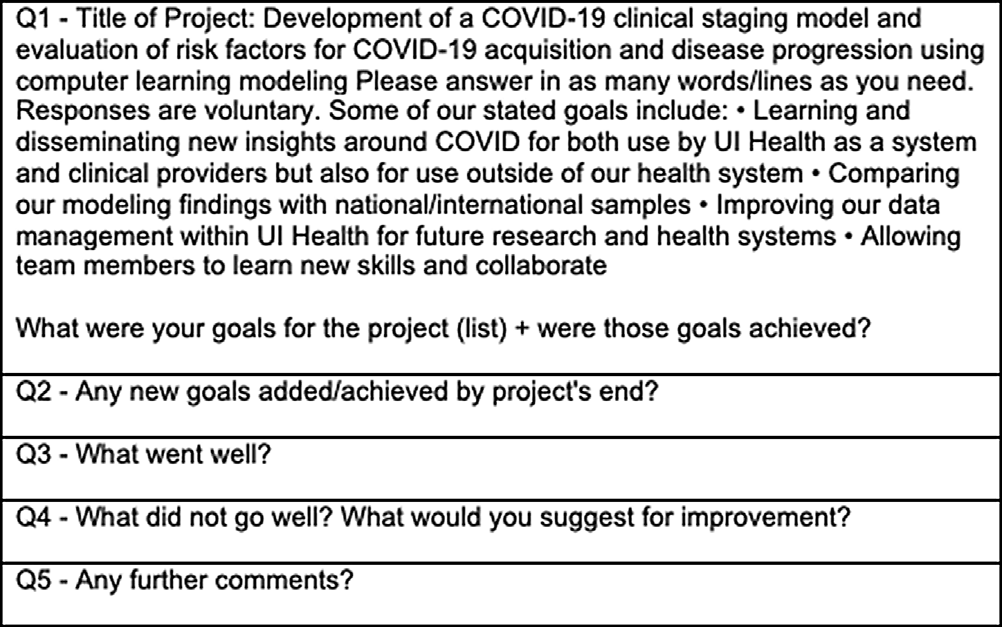
Survey questions.

We qualitatively explored the context, facilitators, and barriers to implementing our COVID-19 clinical staging project. Principle investigators, research staff, and other project team members were invited to complete an anonymous Qualtrics survey (Fig. 1) to reflect on the project. The survey included open-ended questions centering on participants’ opinions about the project, including whether project goals were met, project successes, project failures, and areas that could have been improved. Email invitations to participate were sent in two separate rounds over a month apart. Participation was voluntary and not compensated. Participants provided informed consent prior to completing the survey. In addition, we discussed findings with team members and key representatives of the CCTS. Finally, we conducted a content analysis of surveys and interviews and identified and extracted prominent themes.

## Results

Nine project team members (out of 30 members contacted) completed the survey. While the responses were anonymous, the responders included principal investigators, physicians, engineering faculty, and research staff. The survey responses identified four key themes: Collaboration, Infrastructure, Data Acquisition/Validation, and Model Building.

### Theme 1: Collaboration

One of the primary benefits of this project was the opportunity to foster collaboration and build connections with others within the University of Illinois System, both within and across campuses. The University of Illinois System comprises researchers at different sites throughout the state, including an R1 research university and the primary medical campus in Chicago and an R1 land grant university in Urbana-Champaign. Collaboration was iterative toward shared goals and involved significant amounts of coordination. Typically, such collaboration happens not only between those who represent the same discipline but across disciplines. Throughout the project, we met on a weekly or biweekly basis via Zoom or Webex; these meetings began on 3/20/2020 and provided an opportunity for research groups to share their progress, establish deadlines, identify existing challenges quickly, and ensure that projects remained on track.

Many survey respondents felt satisfied with the amount of collaboration and networking with those at different sites. Our research investigators were generally happy with the progress made and noted their enjoyment of being part of an interprofessional project. However, for some, the collaboration did not go far enough. Weekly meetings often were insufficient to address minor, day-to-day issues. Sometimes there were difficulties within the groups themselves, as is often the case within any larger group project. More frequent technical meetings were needed to address questions regarding implementation-level details not covered at larger, high-level project meetings. How the meetings are conducted is essential, as they must be designed to allow team members to excel, foster a spirit of creativity, and build cohesion toward a mission [15,16]. This process helped the team directly communicate their expectations to one another and ensure the compatibility of their technical approaches in a more up-to-date manner. Some project members felt that the various teams were siloed from one another, with busy clinical content experts not always accessible and technical experts unsure how to best translate between clinical expectations and the practical aspects of their processing and modeling approaches.

With larger teams, it was occasionally challenging to collaborate well with poorly defined goals and consensus aims. Some subgroups felt difficulties in collaboration. Data analysts felt overutilized and left mid-way to work on other operational efforts, partly due to changing priorities and increased responsibilities from the COVID pandemic. As a result, the researchers, physicians, and other informaticists found it challenging to perform adequate data processing. Similar to general healthcare settings, having more precise goals and objectives would have helped provide direction to answer many of our questions [17]. As a result, accountability and responsibility can be promoted among researchers and institutional officers [18].

Having members of two separate University of Illinois System locations (Chicago and Urbana-Champaign) led to a need to work within separate administrative systems. In regards to the regulation of research conduct and ethical approval, administrative operations, and finances, the campuses function as distinct entities, introducing barriers to collaboration and research across campuses.

Communication is essential and notoriously challenging when working between clinicians, researchers, epidemiologists, and data scientists. Available grants often did not engage these groups similarly. NIH grants are often crucial for producing impactful health research [19]. However, attracting funding from the NIH requires understanding the various funding programs [20]. For instance, we noticed separate unique processes to apply for clinical NIH and engineering NSF grants. Salary support for project involvement may also provide motivation and feasibility for greater participation and engagement.

The NLP group required clinician annotators to provide supervised labels for a subset of their data, facilitating automated techniques for extracting relevant clinical variables. Many factors and guidelines exist to develop proper annotation procedures [21]. However, the competing demands on time from other aspects of the project and the ongoing pandemic left the clinicians in the group with little time to provide annotations. As a result, tasks were often deferred, requiring the addition of new team members to IRBs, familiarizing them with project details, and other delays. Thus, requested annotations were rarely completed on time, hindering progress in developing and evaluating NLP models and approaches. A way to ameliorate issues arising from data annotation bottlenecks in future work would be to include room in the budget to pay trained annotators and clinical trainees to complete these tasks, thus incentivizing and protecting the effort and allowing the team to identify annotators able to provide dedicated or protected time for this work [22,23]. Likewise, establishing clear annotation guidelines early in the project, in concert with clinicians, will promote high annotation quality and reproducibility, thereby reducing the need for later potential revision and reannotation. Nondomain and automated annotators supported by machine learning have been considered [24]. Leveraging tools like NLM Scrubber [25] were only more recently utilized on 16 GB of textual clinical note datasets to remove identifiers.

**Lessons Learned**

- Good coordination and iteration toward shared goals are important.
- Frequent meetings help ensure projects stay on track. Even weekly meetings are often insufficient for day-to-day and technical issues that frequently arise.
- Research analysts should be supported in their work, but accountability and responsibility should also be promoted.
- Disparate administrative systems may lead to unforeseen barriers.
- Communication is essential but challenging between clinicians, researchers, epidemiologists, and data scientists. Incentives should be considered for timely participation.

### Theme 2: Infrastructure

The CCTS Biomedical Informatics Core was considered to have provided adequate support, especially when there were questions regarding the data and libraries needed to develop technical methods. Additionally, PACS images were successfully loaded into the CRDW, although with much difficulty due to limited transfer capacity, lack of manual support, and the need to investigate and develop processes to transfer and code images. A secure computing environment (SCE) was created with the University of Illinois’s Advanced Cyberinfrastructure for Education and Research group, which was critical to ensuring patient privacy and security. It was also the first time in our institution’s history that identified patient data was allowed to be placed in such an environment.

The development of a suitable computing infrastructure took considerably longer than expected, partly due to insufficient amounts of RAM, storage capacity, CPU/GPU power, and appropriate data processing software and associated costs. By having multiple projects running concurrently with multiple users, storage space on the SCE quickly ran out, necessitating applications for more space. As a result, the environment became slow, and many applications stalled. In addition, increasing our capabilities required using more of our limited funds. For this reason, the SCE was looked upon unfavorably by our clinicians, researchers, and engineers. It was also considered overregulated and defensively built. One research team member noted that this environment was designed “not at all for the benefit of the scientists… trying to produce results more quickly.” Non-clinician researchers were limited to performing their analyses using only tools approved within the SCE, which hindered them from employing many newer, state-of-the-art tools (e.g., specific machine learning tools and the graphic user interface). Obtaining new software, such as a new data visualization program (e.g., Tableau), required an added layer of information technology management and an additional request. Allowing greater autonomy to the research team, such as a degree of limited sudo access [26], for their software installation within the environment would speed up many processes, including installing text editors and other easily downloadable programs necessary for machine learning.

An additional barrier in the SCE was the absence of GPU support. For technical researchers working with machine learning and NLP models, this prevented them from being able to leverage and experiment with well-known deep learning techniques that may have had a strong performance. Enabling GPU support, in general, would also expedite the processing time for larger machine-learning models, including those that the researchers were able to implement and run using CPUs.

Finally, our imaging team could not use the SCE. Throughout the project, transferring Digital Imaging and Communications in Medicine (DICOM) high-resolution images to the SCE was problematic on many levels. These issues included technical issues and departmental policies regarding the sharing and transferring of data. At the imaging department level, our project was not provided personnel effort to extract DICOM images to transfer to the SCE.

We learned through this process how important it is to work within the structure of our institutional review board (IRB) and CCTS. By nimbly adapting to this workflow, we can hope to be more productive in the future. Much of our research is moving toward cloud computing to mitigate some of these difficulties. Cloud computing will bring unique challenges (e.g., cost containment [27] and security [28]). When doing so, we must continue to commit to protecting health information. Nevertheless, at our institution, we have demonstrated how leveraging cloud computing technology can accelerate public health understanding of emerging health threats [29] and create a compressive synthetic syndromic surveillance system [30]. Utilizing cloud-based systems can provide our COVID-19 team with best practices, implementation guidance, source code, and tools to help cloud deployment and augment our research capabilities. Ideally, federated learning environments will allow multiple health systems to build models without having to share private data [31].

**Lessons Learned**

- Development of novel secured computing environments take time and may lead to unexpected technical challenges.
- When using SCE, elements such as storage space, GPU support, and access control should be managed well.
- SCE should be optimized for desired tasks.
- Cloud computing should be considered to mitigate challenges, but potential cost overruns need to be monitored and managed.

### Theme 3: Data Acquisition/Validation

The research efforts coincided with implementing a new EHR system when UI Health converted from Cerner to Epic in September 2020. Approximately five years of clinical data from Cerner were migrated into Epic. In addition, during our EHR transition over the following year, inconsistencies were compounded in EHR data entry between clinicians while our researchers and data scientists became beholden to how data is entered. For example, data revolving around housing instability and race/ethnicity were not standardized or consistent. Having clinician partners can help elucidate errors and fill in gaps.

Data governance, accountability, and privacy rights remain paramount when overseeing health information. Governmental regulations such as HIPAA, 21st Century Cures Act, and The Common Rule may apply to select research activities. The CCTS has been IRB approved for a CRDW since 2012, and the existing CRDW contains patient data going back to January 2010. All clinical data was ingested into the CRDW and converted to a research common data model (CDM). Prior to September 12, 2020, EHR data was from Cerner. Following that date, EHR data ingested into the CRDW was sourced from Epic. The ACT/i2b2 [32] CDM was chosen as a reasonable common ground for merging data from both EHRs into a research data model format. As needed, the data model has been extended to support various research data requests. While these mappings and the conversion were difficult and time-consuming, the CCTS BI Core has been able to accomplish this seamlessly from the perspectives of most researchers. Some survey respondents noted that the data was received in batches and was not provided within a consistent timeframe. In hindsight, a dedicated pipeline could have been created to update the requested EHR data weekly.

Data transfer was particularly challenging with our imaging informatics efforts. Goals had to be modified over time due to challenges in transferring images. In addition, much paperwork became necessary to obtain information. While the PACS team in the imaging department thought that the PACSGear Media Writer could be used to create and export images to the SCE, it would have resulted in a significant amount of time to individually extract a large amount of DICOM data, given the large cohort. Additionally, IT personnel’s proposed use of a 10-gigabit tunnel for this type of use proved to be too complex for the system to work because of technical issues. Further potential assistance from CCTS to extract the DICOM images was not possible given policy constraints for transfer from the hospital system information systems that could not be changed. Ultimately, concerns were raised at the university campus level. Information Services noted that extraction of PACS images would be complex because of HIPAA concerns and limitations, where direct access to the PACS system is given to researchers.

Eventually, given the inability to extract DICOM images from JPEG, all Chest X-Rays (CXR) were extracted from the PACS system utilizing a scripted method (SikuliX, 2.0.2) and saved in an encrypted laptop as high-quality 24-bit JPEG files (1669 × 1538 to 3032 × 2520 pixels). CXR analysis used for one particular deep learning algorithm predicting comorbidities and clinical outcome details was established in published work [11,12]. A solution to this issue may be to use a web environment with optional de-identification of imaging data to facilitate data distribution within a hospital environment [33]. Additionally, there is an increasing use of enterprise-level PACS systems that use cloud storage for more efficient anonymization and upload [34]. Additionally, GE Healthcare plans to include the Edison Digital Health Platform, which will optimize the use of AI applications across health ecosystems [35]. As a result of these issues and challenges, the CCTS has since added clinical images to its CRDW to help UIC researchers appropriately and securely incorporate these data elements into their future research when needed.

NLP has been used widely in healthcare [36,37] and understanding COVID-19 [38]. The team installed a popular annotation framework, Inception [39], to facilitate annotation on the SCE. While this tool worked well, the notes provided were challenging to utilize consistently, especially given the limited computing resources available in the SCE. The project also suffered from slow progress towards data annotation due mainly to limited annotator availability, proving to be a significant unforeseen bottleneck. Ultimately, the NLP team opted to address this by using techniques designed for low-resource tasks, relying mainly on semi-supervised and active learning methods to identify relevant information in clinical notes.

**Lessons Learned**

- Data standardization, validation, cleaning, and analysis can be a lengthy arduous process.
- Clinician partners can help find errors and fill in gaps.
- Data governance, accountability, and privacy rights remain important when working with a clinical research data warehouse. Data may not be received in a timely or consistent manner.
- Data transfer can be challenging, especially with imaging data.

### Theme 4: Model Building

We successfully created models and compared our results with other published models. These models were published in journals [9–12] and presented at national conferences. However, translation of such models to the clinical setting proved difficult, partly due to the malalignment of incentives throughout our hospital organization. A robust translational process would allow us to accelerate research results in the clinical environment and the community [40]. Roadmaps and frameworks could help to outline investments and activities required to overcome this barrier [41].

We aimed to identify factors associated with poor outcomes in our patients with a new disease for which we had little clinical experience. With a pending EHR transition, models could be considered a clinical decision support tool to improve care and stored outside the EHR. Some hoped to create a point system, similar to Ranson’s criteria [42], placed online with the ability to create shareable plastic reference cards with simple decision rules based on a developed forest tree plot model. There was modest communication between our research team and the hospital system, leading to limited internal dissemination. Getting buy-in became difficult. There is a well-entrenched and reasonable bias toward published models. However, the timeline toward publication is generally slower and not conducive to implementing a new tool in a clinical setting. Because we had an internal grant, went through the IRB, and used the CCTS Bioinformatics Core, there was probably the assumption that it was all research and not operational. Increased support and involvement of clinical informatics physicians could have helped to provide domain expertise, bridge gaps between researchers and clinicians, promote these processes among leadership, and be engaged in change management processes.

Some committee members noted that our models focused primarily on admitted patients, but there was a more general question regarding use in the emergency department and whether the models can help determine whether it was safe to send a COVID-19 patient home. These were valid questions, and to be successful, we would have needed mortality data from neighboring hospitals, the city, and the state. Furthermore, we could have leveraged national data and studies better, including comparing our results to those at other institutions. A proposed COVID-19 registry offered from ACS (American College of Surgery) for use in REDCap [43] was deemed not valuable for data collection efforts. National datasets, like the National COVID Cohort Collaborative (N3C) and the National Technical Information Service managed Limited Access Death Master File (LADMF) [44], were considered. Unfortunately, we did not have the resources to obtain or get up to speed quickly enough on these outside resources to benefit our initial model creation. Eventually, team members participated in some N3C projects, and the acquisition of the LADMF was undertaken to help with future research efforts.

**Lessons Learned**

- Translation of research models to a clinical setting is difficult to achieve.
- Communication with hospital administrators and clinicians is important to obtain buy-in.
- National data sources and registries should be leveraged.

## Conclusion

As an institution, we continue to work on COVID-19 research endeavors. In the process, we learned about our strengths and deficiencies as an organization. We could group these lessons around collaboration, infrastructure, data acquisition/validation, and model-building themes (Table 1). Newer goals did develop out of this project, and we learned more about how our new EHR presents clinical data to our health system. We also continue to work with CCTS to improve our data translation and warehousing capabilities. There has been progress working on analogous efforts in other disease areas, including diabetes, pancreatic cancer, and HIV.

**Table 1.** Lessons learned summary

Theme	Lessons learned
Collaboration	Good coordination and iteration toward shared goals is important. Frequent meetings help ensure projects stay on track. Even weekly meetings are often insufficient for day-to-day and technical issues that frequently arise. Research analysts should be supported in their work, but accountability and responsibility should also be promoted. Disparate administrative systems may lead to unforeseen barriers. Communication is essential but challenging between clinicians, researchers, epidemiologists, and data scientists. Incentives should be considered for timely participation.
Infrastructure	Development of novel secured computing environments take time and may lead to unexpected technical challenges. When using secure computing environments, elements such as storage space, GPU support, and access control should be managed well. Secure computing environments should be optimized for desired tasks. Cloud computing should be considered to mitigate challenges, but potential cost overruns need to be monitored and managed.
Data acquisition/ validation	Data standardization, validation, cleaning, and analysis can be a lengthy arduous process. Clinician partners can help find errors and fill in gaps. Data governance, accountability, and privacy rights remain important when working with a clinical research data warehouse. Data may not be received in a timely or consistent manner. Data transfer can be challenging, especially with imaging data.
Model-building	Translation of research models to a clinical setting is difficult to achieve. Communication with hospital administrators and clinicians is important to obtain buy-in. National data sources and registries should be leveraged.
